# The American and Colonial Journals: Medicine

**Published:** 1838-10

**Authors:** 


### II. THE AMERICAN AND COLONIAL JOURNALS.

### MEDICINE.

### *Case of Pneumonia, with Polypiform Concretion in the Heart.* By Professor DUNGLISON.

THE following case of polypiform concretion proves, with others, that coagula, of the nature of those usually met with in the cavities of the heart on dissection, may form during life, and that the impediment to the circulation through that viscus may be indicated by physical evidences. The semiorganized condition of the concretion was probably owing to the existence of endocarditis or inflammation of the lining membrane of the heart, which facilitated the union between the fibrous portion of the blood and the endocardium or lining membrane of the ventricle; and gave occasion to the semblance at least of organization, which the concretion presented.

Peter Clows, seaman, admitted into the infirmary on the 25th of March. On the 20th, while apparently in perfect health, he was seized with severe pain in the head and vertigo, lasting for some time, and followed by intermittent chills and flushes of heat; great difficulty of breathing, arising, from his account, more from inability to expand the chest than from any particular pain experienced from the attempt. These symptoms continued until his admittance, when his case presented the following appearances. The tongue had a heavy and dark-coloured fur; the eyes were much suffused, and with a yellowish brown tinge; skin hot; great general pain, accompanied with nausea and a considerable degree of languor; breathing very painful, added to a peculiar hitch in respiration; pulse laboured, and very small,—this last symptom arising, no doubt, from the exertion of walking to the house, as in the evening, after some hours' rest, it had become full and bounding; abdomen soft and tender to the touch. Bled on the evening of his entrance, about four o'clock, from a large orifice, to about thirty ounces. Blood, the following morning, presented a thick inflammatory buff. All the symptoms were much relieved by the venesection. As his bowels were very inactive, a powder, composed of fifteen grains of submurias hydrargyri and twenty-five grains of jalap, were administered to him, which brought away, with the most happy effect, a large, dark, and highly offensive stool. As the bleeding of the preceding day had been so beneficial, and as the pain of the head, heat of skin, and difficulty of breathing, still in a measure remained, he was bled, on the morning of the 26th, to about twenty ounces; the blood, after subsidence, also bearing the buffy coat; it was not, however, so heavy as the former. On the application of the stethoscope, the respiratory murmur was totally inaudible in any part of the right lung, very distinct on the left side; percussion on the right gave the dull heavy circumscribed sound of a solid mass; that on the left was hollow and sonorous. The sound on the left side of the heart was so loud as to countenance the idea of dilatation of the cavities with thinness of the parietes; whilst on the right side the sound was indistinct, but there was a peculiar *bruit*, appearing, as it were, compounded of the "bellows' sound" with the *frémissement cataire*, and evidently indicating the existence of some impediment to the circulation of the blood through the right heart. He was put on nauseating treatment; antimonial solution being given every three hours.

As the abdomen was extremely tender to the touch, and as he suffered severely from breathing entirely with the diaphragm, six cupping-glasses were applied, which afforded some slight relief. He complained of a great deal of uneasiness about the heart, which the previous treatment had but slightly benefited. Towards evening, the symptoms became very unfavorable, the anxiety about the præcordium was much increased; pulse very irritable, and so quick as not to be counted; skin covered with a cold clammy sweat; the stupor and coma, which from the first had been prominent symptoms in the case, were much aggravated. In short, he was evidently dying; stimulants were diligently administered through the night, but without effect; he died next morning at eleven o'clock.

*Necroscopy*, four hours after death. Skin extraordinarily hot. On examining the chest, the right lung was found twice as large as in the healthy state, and completely hepatized throughout its whole extent. On cutting into it, a thin muco-purulent matter exuded from every part of it. The left lung, though exhibiting some marks of inflammation, was otherwise healthy throughout. On taking out the heart, the right pulmonary artery was found completely plugged up by a semi-organized mass, having the appearance of muscle, and extending through the semilunar valves, and attaching itself strongly to the columnæ carneæ of the right ventricle. Whether this production was caused by the engorgement of the lung, thus giving rise to a remora of the blood and deposition of fibrine, I do not pretend to say; it is, however, an interesting fact, and may throw some light on the nature of polypus of the heart. From the symptoms and auscultation during life, and its organized appearance, it certainly could not have been formed during the last struggle. *American Medical Intelligencer. July* 1, 1837.

